# Geospatial Variation in Vaccination Coverage and Zero-Dose Prevalence at the District, Ward and Health Facility Levels Before and After a Measles Vaccination Campaign in Nigeria

**DOI:** 10.3390/vaccines12121299

**Published:** 2024-11-21

**Authors:** C. Edson Utazi, Iyanuloluwa D. Olowe, H. M. Theophilus Chan, Winfred Dotse-Gborgbortsi, John Wagai, Jamila A. Umar, Sulaiman Etamesor, Brian Atuhaire, Biyi Fafunmi, Jessica Crawford, Adeyemi Adeniran, Andrew J. Tatem

**Affiliations:** 1 WorldPop, School of Geography and Environmental Science, University of Southampton, Southampton SO17 1BJ, UK; i.d.olowe@soton.ac.uk (I.D.O.); w.dotse-gborgbortsi@soton.ac.uk (W.D.-G.); a.j.tatem@soton.ac.uk (A.J.T.); 2 Department of Statistics, Nnamdi Azikiwe University, Awka PMB 5025, Nigeria; 3 School of Mathematical Sciences, University of Southampton, Southampton SO17 1BJ, UK; hmtc1u18@soton.ac.uk; 4 World Health Organization Consultant, Abuja, Nigeria; johnwagai@gmail.com; 5 National Primary Health Care Development Agency, Abuja, Nigeria; jamila.abubakar@nphcda.gov.ng (J.A.U.); sulaiman.etamesor@nphcda.gov.ng (S.E.); 6 Gavi, The Vaccine Alliance, Geneva, Switzerland; brianatu@gmail.com (B.A.); jcrawford@gavi.org (J.C.); 7 National Bureau of Statistics, Abuja, Nigeria; biyifafunmi@nigerianstat.gov.ng (B.F.); saadeniran@nigerianstat.gov.ng (A.A.)

**Keywords:** measles vaccination, zero dose, post-campaign coverage survey, Bayesian geostatistical model, health facility catchment areas

## Abstract

Many measles endemic countries with suboptimal coverage levels still rely on vaccination campaigns to fill immunity gaps and boost control efforts. Depending on local epidemiological patterns, national or targeted campaigns are implemented, following which post-campaign coverage surveys (PCCSs) are conducted to evaluate their performance, particularly in terms of reaching previously unvaccinated children. Due to limited resources, PCCS surveys are designed to be representative at coarse spatial scales, often masking important heterogeneities in coverage that could enhance the identification of areas of poor performance for follow-up via routine immunization strategies. Here, we undertake geospatial analyses of the 2021 measles PCCS in Nigeria to map indicators of coverage measuring the individual and combined performance of the campaign and routine immunization (RI) at 1 × 1 km resolution and the ward and district levels in 13 states. Using additional geospatial datasets, we also produced estimates of numbers of unvaccinated children during the campaign and numbers of measles-containing vaccine (MCV) zero-dose children before and after the campaign at these levels and within health facility catchment areas. Our study revealed that although the campaign reduced the numbers of MCV zero-dose children in all the districts, areas of suboptimal campaign and RI performance with considerable numbers of zero-dose children remained. Our analyses further identified wards and health facility catchment areas with higher numbers of unvaccinated children within these areas. Our outputs provide a robust evidence base to plan and implement follow-up RI strategies and to guide future campaigns at flexible and operationally relevant spatial scales.

## 1. Introduction

Vaccination remains one of the most successful public health interventions, averting an estimated 3.5 to 5 million vaccine-preventable deaths every year [1]—a substantial improvement since the introduction of the World Health Organization Expanded Programme on Immunization (WHO EPI) in 1974 [2]. However, vaccination coverage remains suboptimal in many places [3]—a situation further exacerbated by the COVID-19 pandemic with major disruptions to immunization and other health services [4,5], although gradual recovery is being made [6]. Within the past few decades, there have been concerted efforts to improve global vaccination coverage levels, which have generally resulted in improved access to vaccines and decreases in various kinds of inequities [7]. These efforts are shaped and driven by global policy frameworks such as the United Nations’ Sustainable Development Goals (SDGs) [8], the WHO’s Immunization Agenda 2030 [7], Gavi, the Vaccine Alliance’s Strategy 5.0 [9], the Global Vaccine Action Plan [10] and Reaching Every District [11,12]. Together, these initiatives and strategies emphasize the importance of strengthening immunization systems to reach zero-dose and missed communities and have set ambitious targets at the global, national and subnational (e.g., the district level) levels to reduce vaccine preventable diseases.

Indicators of vaccination coverage are crucial to monitor programme performance, understand geographical and other inequities in coverage, and measure the effectiveness of interventions to increase coverage, such as supplementary immunization activities (SIAs) or vaccination campaigns. Estimates of indicators of vaccination coverage can be obtained from administrative/official sources or vaccination coverage surveys, such as a post-campaign coverage surveys (PCCSs) used to monitor the performance of vaccination campaigns. Administrative estimates, though available at different levels of the health system and useful for near real-time monitoring, are often inaccurate in many countries due to numerator and denominator errors [13,14]. Vaccination coverage surveys, on the other hand, are mostly designed to produce estimates at the national and provincial levels (or administrative level one areas) due to the high cost of more intensive sampling to produce estimates at more granular levels. Due to the increasing need for more accurate spatially detailed estimates to support programme planning, implementation and monitoring, particularly at the district level, geospatial modelling approaches that leverage existing survey data and spatial relationships between indicators of coverage and geospatial covariates have now become popular [15,16,17,18]. In the context of vaccination campaigns, geospatial analysis of PCCS data can be used to produce district and ward level estimates [19,20] which can be compared with campaign administrative estimates or tally sheet data to better understand campaign performance and highlight poor performing areas more precisely. Modelled coverage estimates can be integrated with other datasets such as gridded population data to highlight areas with relatively higher numbers of zero-dose children at the end of a campaign to guide the implementation of follow-up routine immunization (RI) strategies [20].

Measles remains endemic in Nigeria, with 10,649 and 23,983 cases reported to WHO in 2021 and 2022, respectively [21]. Recent reports also indicate continued occurrence of measles cases throughout the country [22], especially among children aged under 5 years. WHO and UNICEF estimate that the routine coverage of the first and second doses of the measles vaccine in Nigeria were 60% and 38% in 2021, respectively, and have remained the same in 2023 [3], although 95% coverage with two doses is required to interrupt measles transmission [23]. The latest WUENIC estimates also showed that Nigeria is one of the countries with the highest numbers of MCV zero-dose children [6], i.e., those who had not received the first dose of MCV. Due to its suboptimal RI programme (risk factors for non-vaccination are well-studied elsewhere [24]), Nigeria implements national follow-up measles campaigns targeting children aged 9–59 months approximately every two years, following a catch-up measles vaccination campaign in 2005–2006. A PCCS is often implemented at the end of each campaign to estimate the coverage of different indicators of campaign performance [25]. Some of the recent measles campaigns prior to the 2021 campaign are the 2017–2018 national campaign which had a PCCS coverage estimate of 88% and the 2019 campaign implemented in 20 northern states, where higher risks of outbreaks were identified, with a PCCS estimate of 89%. The persistence of measles outbreaks despite these repeated vaccination campaigns is highly indicative of suboptimal performance of vaccine delivery strategies and calls for more robust scientific evidence to support the combination of these strategies for more effective disease control.

In previous work [19,20], we have undertaken geospatial analyses of the 2017–2018 and 2019 measles PCCS in Nigeria to map the coverage of indicators of campaign performance. Our goal in the current work is to analyze the 2021 Nigeria measles PCCS to produce coverage estimates and corresponding estimates of unvaccinated children at 1 × 1 km resolution and the district level. We also aim to produce estimates of numbers of MCV zero-dose or unvaccinated children within health facility catchment areas and at the ward level before and after the campaign, to support further evaluation of the impact of the campaign and the design and implementation of post-campaign vaccination strategies via the RI programme, especially in regard to establishing a linkage between residual zero-dose cases and the health system.

## 2. Methods

### 2.1. 2021 Measles Post-Campaign Coverage Survey Data

As part of its measles control and elimination efforts, Nigeria planned a nationwide measles campaign in 2021. However, due to logistical challenges, the campaign was implemented in only 13 states, namely Kaduna, Kano, Kwara, Katsina, Yobe, Taraba, Borno, Abia, Imo, Ebonyi, Bayelsa, Sokoto and Kebbi—see Supplementary Figure S1. These states were prioritized based on the estimated risk of measles outbreaks as determined by the estimated herd immunity. The campaign was implemented between November and December 2021 [25], following which a PCCS was implemented in each of the 13 states between January and February 2022. A two-stage sampling procedure was used in the PCCS, which involved the selection of enumeration areas (EAs) (i.e., the survey clusters) from a national sampling frame and conducting household listing in each selected EA. During this stage, some areas (e.g., some LGAs in the northeast and southeast) that were inaccessible to the survey teams due to insecurity were excluded from the sampling frame. A systematic random sampling technique was used in the second stage to select households to be interviewed in each selected EA. A total of 15 households were selected from each of the 40 EAs in the 13 states and all children in the target age group (i.e., 9–59 months) in these households were eligible to be enrolled in the survey. This yielded a total of 7800 households across all the 13 states.

**Figure 1:**
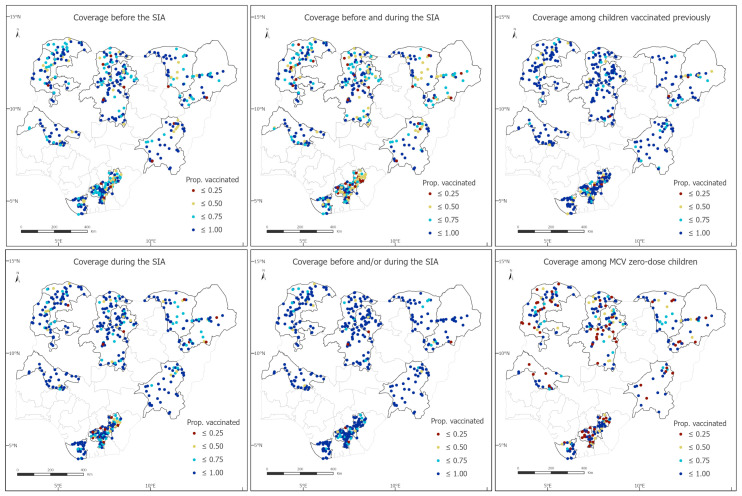
2021 Measles post-campaign coverage survey cluster-level vaccination coverage data for children aged 9–59 months.

For each child included in the survey, we obtained information about their age (in months) and the displaced geographical coordinates of the cluster from which they were sampled, alongside other relevant information. The cluster coordinates were displaced to protect the confidentiality of the respondents following a similar procedure described in Perez-Haydrich et al. [26]. Data on MCV coverage before the campaign were obtained from home-based records (HBRs) of routine vaccination or through parental/caregiver recall. Additionally, for campaign-related coverage, evidence of vaccination included finger-marks. Based on these data and following the methodology used in previous analyses [19], we analyzed six indicators (Figure 1) assessing the individual performance of the campaign and its combined performance with routine immunization for children aged 9–59 months.

These indicators are: (i) coverage before the SIA/campaign, (ii) coverage during the SIA, (iii) SIA coverage among zero-dose children, (iv) SIA coverage among children vaccinated previously, (v) coverage before and during the campaign (i.e., coverage with at least two doses of MCV by the end of the campaign) and (vi) coverage before and/or during the campaign (i.e., coverage with at least one dose of MCV by the end of the campaign). Whilst indicators (ii)–(iv) measure the performance of the current campaign, indicators (i), (v) and (vi) assess the combined effect of RI and campaigns (including previous campaigns). Detailed definitions of these indicators are provided in Utazi et al. [19].

The cluster level vaccination coverage data for each of the six indicators are mapped in Figure 1. A total of 5528 children aged 9–59 months were sampled during the survey. Out of these, 5526 and 5486 children had complete records (i.e., known vaccination status) for coverage before the campaign and coverage during the campaign, 4221 (76%) and 4532 (83%) of whom were vaccinated, respectively, across the 13 states. For all the indicators, cluster level sample sizes ranged from 1 to 35 children—see Supplementary Figure S2. The figure shows that cluster level sample sizes are lowest for coverage among MCV zero-dose children compared to other indicators. As in previous work [19], we excluded clusters where one child was surveyed during model-fitting as this often results in higher prediction uncertainty.

### 2.2. Geospatial Covariate Data, Population Data and Covariate Selection

Following previous work [17,18,19,27,28], we assembled a suite of geospatial covariate information for our analysis. This included remoteness (e.g., distance to roads and travel time to the nearest health facility), socioeconomic (e.g., nightlight intensity, livestock density) and environmental (e.g., elevation, temperature and precipitation) variables—see, e.g., Supplementary Table S1. These covariates help improve the prediction of vaccination coverage and their consideration was informed by inclusion in previous studies [17,18,19,27,28] and direct or proximate associations with vaccination coverage. We ensured the alignment of the temporally varying covariates with the reference period of the survey wherever possible, and in other cases, we obtained covariate data closest to the survey year. These covariates were processed as detailed in previous work [18,28] to produce 1 × 1 km raster layers and cluster-level data using the (displaced) geographical coordinates from the 2021 PCCS. Furthermore, we also obtained gridded population data for children aged 12–59 months and under 5 years old in 2021 from WorldPop [29]. These were used in our work to calculate aggregate estimates of coverage and estimates of numbers of MCV zero-dose and/or unvaccinated children.

We undertook covariate selection using approaches similar to those outlined in Utazi et al. [19]. For each of the three directly modelled indicators (see modelling section), the covariate selection process involved checking the relationships between the covariates and vaccination coverage on the logit scale and applying the log transformation to the covariates where necessary; fitting of single covariate models and ranking the covariates based on their predictive ability (e.g., as determined using predictive R^2^ values); checking for multicollinearity and choosing between highly correlated covariates (correlation > 0.8 or variance inflation factor > 4.0) using their ranks; and using stepwise regression (backward elimination based on Akaike Information Criterion) to select the best model/combination of covariates for modelling the indicator. The steps described above were implemented in a non-spatial framework using binomial regression models. We created a uniform set of covariates for all three modelled indicators from the covariates selected for each indicator (Supplementary Table S1 and Supplementary Figure S3). This mainly included covariates that were significant in at least two of the three best models.

### 2.3. Health Facility, Administrative Boundary and Building Footprint Data

Administrative levels one (states) and two (districts) digital boundary files (shapefiles) for Nigeria were obtained from WHO [30]. We also obtained ward level and additional administrative boundaries from GRID3 [31], as well as detailed data on the locations of health facilities for Ebonyi and Kano states. Both states were selected for illustration purposes only. The health facility data included their geocoordinates, functional status, category (general hospital, private clinic, maternity home, etc.), type (primary, secondary or tertiary) and ownership (private, public, missions/religious, etc.). For Kano state, the data originally comprised 1613 facilities, whereas for Ebonyi, 1177 facilities were obtained. Upon further validation and consultation with local health officials in Kano state, the number of facilities was reduced to 260. For Ebonyi, the data were filtered to include health facilities likely to offer vaccination services based on expert knowledge, yielding 418 facilities (see Supplementary Figure S4). To enable the delineation of the catchment areas of the health facilities, friction raster layers at walking and motorized travel scenarios were obtained from https://malariaatlas.org/project-resources/accessibility-to-healthcare/ (accessed on 28 February 2024) These friction raster layers are 1 × 1 km datasets produced by Weiss et al. [32] using a combination of geographic factors, with each pixel representing the cost of movement (that is, time in minutes) required to travel per 1 km.

To demonstrate the utility of our coverage maps and corresponding zero-dose estimates for developing microplans to support intervention strategies targeting zero-dose children following vaccination campaigns, we also obtained building footprint data from Google [33].

### 2.4. Bayesian Geostatistical Model, Model Fitting and Prediction

To model and predict vaccination coverage at 1 × 1 km resolution using cluster-level PCCS data, we employed a Bayesian geostatistical modelling framework. Let $Z\left(s_{i}\right)$ denote the number of children vaccinated at survey location $s_{i}\;\left(i=1,\ldots ,n\right)$ and $m\left(s_{i}\right)$ the number of children sampled at the location. The first level of the geostatistical model assumes that

(1) $$Z\left(s_{i}\right)|p\left(s_{i}\right)∼\mathrm{Binomial}\left(m\left(s_{i}\right),p\left(s_{i}\right)\right),$$

where $p\left(s_{i}\right)\;\left(0\leq p\left(s_{i}\right)\leq 1\right)$ is the true vaccination coverage at location $s_{i}.$ We model $p\left(s_{i}\right)$ using the logistic regression model as

(2) $$\mathrm{logit}\left(p\left(s_{i}\right)\right)=x{\left(s_{i}\right)}^{T}\beta +\omega \left(s_{i}\right)+\gamma \left(s_{i}\right)$$

where $x\left(s_{i}\right)$ is the vector of covariate data associated with location $s_{i}$ , β is a vector of the corresponding regression coefficients, $\gamma \left(s_{i}\right)$ is an independent and identically distributed (iid) Gaussian random effect with variance, $\sigma_{\gamma }^{2}$ , used to model non-spatial residual variation, and $\omega \left(s_{i}\right)$ is a Gaussian spatial random effect used to capture residual spatial correlation in the model. That is, $\omega ={\left(\omega \left(s_{1}\right),\ldots ,\omega \left(s_{n}\right)\;\right)}^{T}∼N\left(0,\;\Sigma_{\omega }\right)$ , where $\Sigma_{\omega }$ is assumed to follow the Matérn covariance function [34] given by $\Sigma_{\omega }\left(s_{i},\;s_{j}\right)=\frac{\sigma^{2}}{2^{\nu -1}\Gamma \left(\nu \right)}{\left(\kappa ∥s_{i}-s_{j}∥\right)}^{\nu }\;K_{\nu }\;\left(\kappa ∥s_{i}-s_{j}∥\right)$ . The notation ∥.∥ denotes the Euclidean distance, $\sigma^{2}>0$ is the marginal variance of the spatial process, κ is a scaling parameter related to the range $r\left(r=\frac{\sqrt{8\nu }}{\kappa }\right)$ —the distance at which spatial correlation is close to 0.1, and $K_{\nu }$ is the modified Bessel function of the second kind and order ν>0 . For identifiability reasons, we set the smoothing parameter, ν=1 , see [35].

We fitted the model for the three indicators (i) coverage before the SIA (ii) SIA coverage among MCV zero-dose children and (iii) SIA coverage among children vaccinated previously, to ensure the internal consistency of the six PCCS indicators as detailed in previous work [19]. The modelled 1 × 1 km estimates for the remaining three indicators, namely coverage during the campaign, coverage before and during the campaign and coverage before and/or during the campaign, were then calculated using samples from the posterior predictive distributions of these directly modelled indicators.

In each case, the model described in Equations (1) and (2) was fitted in a Bayesian framework using the integrated nested Laplace approximation—stochastic partial differential equation (INLA-SPDE) approach (INLA-SPDE) approach [35,36]. Aggregate (district and state) level predictions were obtained as population-weighted averages taken over the 1 × 1 km grid cells falling withing each administrative area. Also, estimates of numbers of unvaccinated children before and during the campaign were obtained by integrating the coverage maps with gridded population data as discussed previously and aggregating these to relevant administrative levels.

Approaches to evaluate the out-of-sample predictive performance of the fitted model are discussed elsewhere [19,27]. Here, we rather focus on exploring the patterns seen in the estimates produced by these models. All analyses were carried out using the R programming language version 3.4.3 [37] and the R-INLA package [38].

### 2.5. Methodology for Health Facility Catchment Area Delineation

We delineated the catchment areas of the health facilities in Kano and Ebonyi states using the *r.cost* tool in QGIS software version 3.34.12 [39,40]. The tool utilized the locations of the health facilities and cost/friction raster layers at walking and motorized travel scenarios described previously. Based on these input datasets, it obtained a cumulative cost raster representing the total cost to reach the nearest health facility from each 1 × 1 km grid cell. It then identified grid cells that were within a certain “cost” or travel time of a health facility and used these to delineate its catchment area. We then overlaid the estimated catchment areas on the grid level zero-dose estimates to calculate the numbers of zero-dose children living within these areas. We also demonstrated how areas for fixed and outreach services could be determined within the catchment areas through using building footprint data.

## 3. Results

### 3.1. 1 × 1 km and District Level Estimates of PCCS Indicators

**Figure 2:**
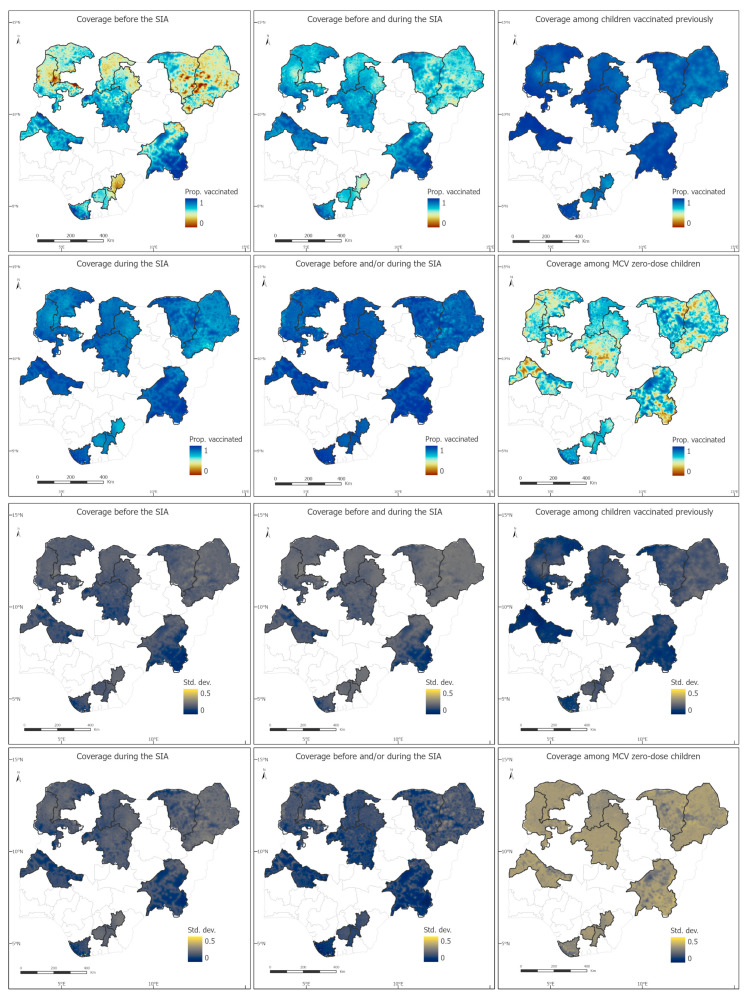
1 × 1 km modelled estimates of coverage among children aged 9–59 months for six measles post-campaign coverage survey (PCCS) indicators and associated uncertainty estimates shown as standard deviations.

We estimated substantial heterogeneities in the spatial distributions of the PCCS indicators as shown in Figure 2 and Supplementary Figure S5, although these seemed relatively less pronounced in the southern states of Abia, Ebonyi and Imo, and in the indicators: coverage during the campaign, coverage before and/or during the campaign and coverage among children vaccinated previously. Areas of lower coverage before the campaign appear to be concentrated in the northeastern and northwestern states of Borno, Yobe, Kebbi, Sokoto, Katsina and Kano (eastern part of the state), and Ebonyi state in the southeast. We estimated that, overall, coverage during the campaign, compared to coverage before the campaign, was greater in all the states except Imo and Kaduna states (the direct survey estimates showed similar patterns in both states as well as Borno state [24]). Coverage during the campaign was also estimated to be relatively lower in all three southeastern states (Abia, Ebonyi and Imo), Borno and parts of Kano, Kebbi, Yobe and Sokoto states.

Our analyses also revealed interesting patterns in the performance of the campaign in terms of reaching previously vaccinated and zero-dose children. We estimated that in all 13 states, the campaign generally reached more previously vaccinated children than zero-dose children. The greatest differences between both indicators were observed in the more populous states of Kano and Kaduna, whereas the least differences were observed in Abia, Bayelsa and Ebonyi. Five states with the lowest coverage among zero-dose children were Kaduna, Kano, Borno, Kebbi and Sokoto, although there are substantial heterogeneities in this indicator in Kwara, Taraba and Yobe states (Figure 2 and Supplementary Figure S5), where apparent areas of low coverage can be seen.

Interestingly, when considering coverage with at least one dose of MCV (i.e., coverage before and/or during the campaign), we generally observed higher coverage levels compared to coverage before the campaign. This is particularly evidenced at the state level where we estimated higher coverage levels for this indicator in all the states compared to coverage before the campaign (Supplementary Table S2). This demonstrates that although coverage during the campaign did not exceed pre-campaign coverage in all the states, enough of previously unvaccinated children were reached in many areas to boost coverage after the campaign beyond the levels seen before the campaign at the state level. Furthermore, coverage with at least one dose has less spatial heterogeneities than coverage with two doses. In the latter case, there are substantial areas of low coverage in all the states except Kwara, Bayelsa and much of Taraba state.

The uncertainties associated with the grid level estimates are very similar across the indicators, although these are slightly lower for coverage before the campaign. The spatial distributions of the uncertainty estimates appear to be mostly affected by the distribution of the corresponding coverage estimates (estimates close to the endpoints on the unit interval are less uncertain than estimates close to the midpoint—an artefact of the binomial distribution used to model the data), rather than the spatial coverage/distribution of the input data.

**Figure 3:**
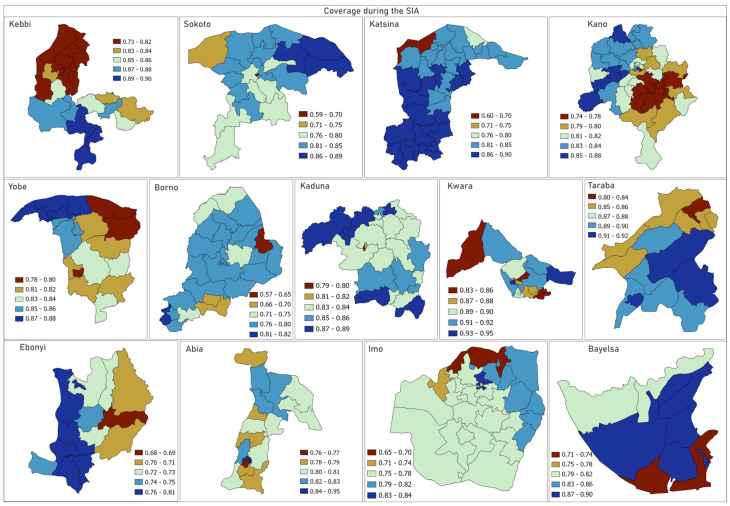
Local government area (LGA) level estimates of coverage during the campaign among children aged 9–59 months.

In Figure 3 and Supplementary Figure S6, we further explore the distribution of coverage during the campaign and coverage among zero-dose children at the LGA level, with a view to highlighting LGAs with particularly lower coverage levels for these indicators in each state. For coverage during the campaign, we observe that LGA coverage is most variable in Bayelsa state and least variable in Kaduna state. We estimated that in Kano, 17 LGAs (out of 44) had coverage levels below 80% whereas in Borno, 26 (out of 27) LGAs had campaign coverage below 70%. In Ebonyi state, LGAs with coverage less than 72% include Abakaliki, Ebonyi, Ikwo and Izzi. For coverage among zero-dose children, our analyses revealed that all the LGAs in Kaduna, for example, had <60% coverage for this indicator, despite having 79–89% overall campaign coverage. In Abia, a relatively higher performance was seen, with coverage among MCV zero-dose children ranging between 63% and 82% at the LGA level. Similar patterns can be observed in the other states.

### 3.2. Estimates of Numbers of Zero-Dose Children Before and After the Campaign at the LGA Level

**Figure 4:**
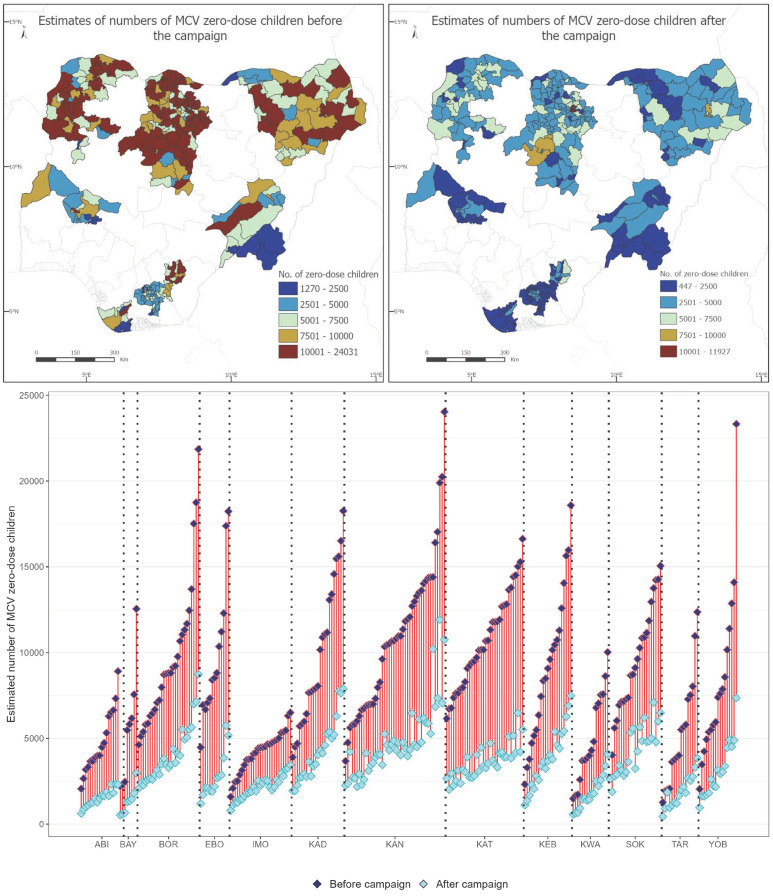
Estimates of numbers of zero-dose children aged 12–59 months before and after the 2021 measles vaccination campaign at the LGA level.

We produced estimates of numbers of zero-dose children before (estimated using coverage before the campaign) and after (estimated using coverage before and/or during the campaign) the campaign to further evaluate the impact of the campaign in terms of reducing the numbers of MCV zero-dose children. Figure 4 shows that the campaign had the most impact in Kano and Katsina states, both of which had the largest estimated number of children aged 12–59 months, although considerable numbers of zero-dose children remained after the campaign in some LGAs in both states. There are also some LGAs in Borno, Kebbi, Sokoto and Yobe states which experienced large reductions in numbers of zero-dose children after the campaign. LGAs with the lowest reductions in the numbers of zero-dose children are mostly concentrated in Abia and Imo states, both of which had among the lowest populations of children aged 12–59 months. The figures also reveal that considerable numbers of zero-dose children remained after the campaign particularly in areas where there were relatively higher numbers of zero-dose children before the campaign. LGAs where >5000 MCV zero-dose children aged 12–59 remained after the campaign are mostly concentrated in the northern states of Borno (e.g., Jere, Bama and Gwoza), Kaduna (e.g., Igabi, Chikun and Zaria), Kano (e.g., Kumbotso, Ungogo and Gwale), Kebbi (e.g., Jega, Wasagu-Danko and Birnin Kebbi) and Sokoto (e.g., Dange Shuni, Tambuwal and Kware). Two LGAs in Ebonyi state (Izzi and Ikwo) also had >5000 MCV zero-dose children after the campaign.

### 3.3. Estimates of Numbers of Unvaccinated Children During the Campaign at the Ward and Health Facility Catchment Area Levels

**Figure 5:**
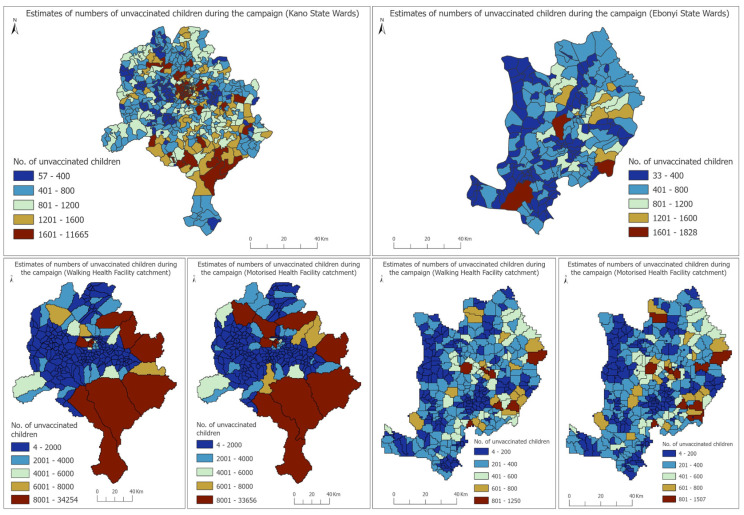
Estimates of numbers of unvaccinated children aged 12–59 months at the ward and health facility catchment area levels during the campaign.

At the ward and health facility catchment area levels, we show the spatial distribution of estimates of numbers of unvaccinated children during the campaign (estimated using coverage during the campaign—includes zero-dose and previously vaccinated children) in Kano and Ebonyi states in Figure 5, as examples. In Kano state, we estimated that 98 (out of 484) wards had >1200 unvaccinated children, whereas in Ebonyi, there were 9 of such wards. In Kano, the wards with the most numbers of unvaccinated children (>8000) during the campaign (Dorayi, Rijiyar Zaki, and Kabuga wards) are also located in LGAs where we had estimated higher numbers of MCV zero-dose children after the campaign. In Ebonyi, this is also the case in Ndiegwi Inyimegu, Ndiewgu Echara 1 and Ndietta wards, with >1500 unvaccinated children during the campaign.

At the health facility level, we also highlight catchment areas created using both walking and motorized travel times where relatively higher numbers of unvaccinated children during the campaign were estimated. Our results generally show strong similarities in the spatial distributions of the unvaccinated children obtained through using both travel time catchment areas (although we found differences in the distributions of numbers of children living within different travel time bands estimated using both travel times, see Supplementary Figures S7 and S8). We, therefore, focus more on the trends observed in the walking travel time catchment areas. In Kano, health facilities with relatively higher numbers of unvaccinated children in their catchment areas (>15,000 children) during the campaign include Kibiya Primary Health Center (PHC), Darki PHC, Chiranci Basic Health Center and Yarganji Health Post, some of which are located within LGAs where we had estimated larger numbers of MCV zero-dose children after the campaign. In Ebonyi state, health facilities with relatively higher numbers of unvaccinated children in their catchment areas (>800 children) during the campaign include Onuebonyi Health Center, Onu Nwakpu Health Center, Echara Health Center, Ebonyi State University Teaching Hospital and Omege Umuezeokoha Primary Health Center. Estimates of numbers of MCV zero-dose children living within these catchment areas can also be straightforwardly estimated if needed.

### 3.4. Identification of Areas for Fixed and Outreach Services Within Health Facility Catchment Areas Using Building Footprint Data

Identifying, reaching and vaccinating zero-dose children or those missed during a vaccination campaign require not only spatially detailed data regarding their sizes but also detailed knowledge of settlements/buildings/households where they reside to enable, for example, house-to-house delivery of vaccination services or the location of temporary vaccination posts. Microplans for various interventions are often based on these detailed datasets.

**Figure 6:**
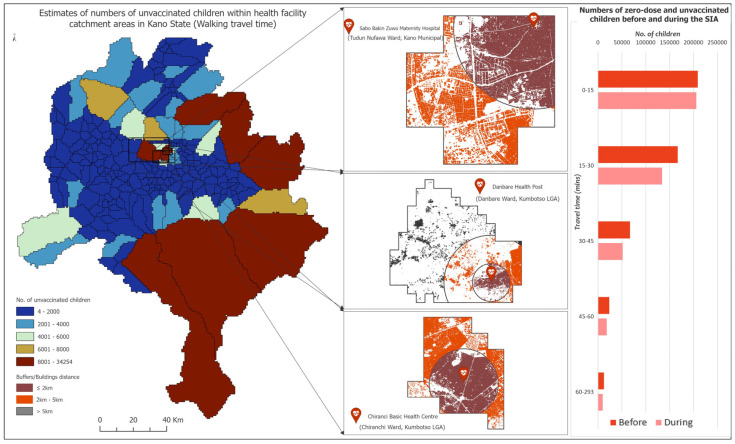
(**Left panel**) Estimates of numbers of unvaccinated children during the campaign at the health facility catchment area level in Kano state. Insets show locations of the corresponding health facilities, and the buildings identified within the catchment areas through using Google building footprint data. (**Right panel**) Estimates of numbers of unvaccinated children before and during the campaign within different walking travel time bands in the state.

In Figure 6, we show the distribution of buildings within the catchment areas of some health facilities where we estimated some of the highest numbers of unvaccinated children during the campaign in Kano. We also show areas within the catchment areas of example health facilities which are within 2 km, 2–5 km and >5 km of the health facilities and are often designated as areas for fixed, outreach and mobile services during field work [41]. Within the catchment area of Sabo Bakin Zuwo Maternity Hospital, we identified a total of 90,636 buildings, of which 65,153 were located within 2 km of the health facility. For Danbare Health Post and Chiranci Basic Health Centre, we identified 93,139 and 169,085 buildings, of which 18,547 and 110,746, respectively, were located within 2 km of these health facilities.

Additionally, when comparing estimates of numbers of unvaccinated children during the campaign within different walking travel time bands, Figure 6 (right panel) shows that greater numbers of these children lived closer to the health facilities (potentially due to their location in more densely populated areas), highlighting their centrality to reaching zero-dose and under-immunized children within the country. The same pattern was also seen when using the motorized catchment areas, except that the numbers declined dramatically with increasing travel times (Supplementary Figures S7 and S8).

To further support field activities, the catchment areas and wards shown here can be further subdivided to create enumeration area type units with manageable numbers of buildings which can be covered by small-sized field teams within a specified period of time using, e.g., the preEA tool [42]. Detailed maps of these enumeration areas as shown in Supplementary Figure S10 can also be produced.

## 4. Discussion

Evaluating the spatial distribution of indicators of vaccination coverage and corresponding estimates of residual un/under-vaccinated children following a measles vaccination campaign is crucial to understanding the performance of the campaign and its combined performance with previous campaigns and routine immunization, particularly in terms of reaching zero-dose children and ensuring full immunization with the recommended two doses of the measles vaccine. We mapped six PCCS indicators and produced estimates of numbers of zero-dose/unvaccinated children before, during and after the campaign at resolved spatial scales, namely the district, ward and health facility catchment areas, to provide operational outputs that will help programme managers design and implement effective follow-up RI strategies in all areas of interest. These analyses are highly relevant considering the geographical inequities that exist in coverage at these lower levels and the evidence that the campaign did not reach the target coverage of 95% (with two doses) in all 13 states, which is required to interrupt measles transmission and prevent outbreaks [23].

As we have noted previously, administrative estimates of campaign coverage, though available at detailed spatial scales, suffer from some limitations. Our modelled estimates can therefore be used for data quality checks when compared with these at the LGA and ward levels to ascertain the likely coverage in these areas where implausible administrative coverage estimates (e.g., those >100%) had been obtained. This comparison would not have been possible using the direct survey estimates since the survey was not designed to be representative at the LGA level. We compared our modelled estimates with direct survey estimates at the state level and observed strong correlations between these, with the differences being less than 5% in most cases (Supplementary Table S2, Supplementary Figure S9 and [25]). However, in Borno state, we estimated 14% lower and 15% higher coverage before and during the campaign, respectively, which is likely due to poor spatial coverage of the survey clusters in the state as a result of insecurity (Figure 1). Also, for Imo, we observed a difference of 19% between our modelled estimate and the direct survey estimate for coverage during the campaign. We investigated this further and found that our modelled estimate (77%) was nearly the same as the unweighted estimate (78.7%) for the state, meaning that the observed difference is likely due to the weighting used to produce the direct estimate for this indicator for the state. Our modelled outputs can therefore enhance data quality checks and guarantee the availability of high-quality data for programming.

Our analyses showed that considerable numbers of zero-dose children (>5000) aged 12–59 months remained after the campaign in LGAs where there were substantially higher numbers of zero-dose children before the campaign. These LGAs were mostly located in the northeast and northwest, corroborating findings in previous studies [16,20,43]. The greatest reductions in the numbers of zero-dose children after the campaign were observed in states with the largest estimated numbers of children aged 12–59 months as expected, but there were some areas (e.g., two LGAs in Ebonyi) in less populated states where large reductions were also observed. We also found that in the example states of Kano and Ebonyi, wards with relatively higher numbers of unvaccinated children during the campaign were located in LGAs where we also estimated higher numbers of zero-dose children after the campaign, highlighting the importance of prioritizing reaching previously unvaccinated children during campaigns. A similar pattern was also observed at the health facility catchment area level. The persistence of these clusters of susceptibility is likely to sustain disease transmission and potentially attenuate the outcome of the campaign [44]. The analyses presented here should therefore be routinized, particularly where targeted coverage levels are not reached during vaccination campaigns, to guide the deployment of effective, targeted follow-up RI strategies in all problematic areas. This will also make for a more effective combination of both delivery mechanisms to support measles control efforts in the country and other endemic settings.

Nigeria introduced the second dose of the measles vaccine (MCV2) in late 2019 in the southern zones and in 2020 in the northern zones. The latest 2023 WUENIC estimates [3] indicate that MCV2 coverage had remained at 38%, which is the same as the 2020 coverage. While efforts are being made to reach MCV zero-dose children through campaigns and RI strategies, some effort should also be dedicated to ensuring that full immunization is attained in areas where RI coverage has improved consistently. These areas can be identified by triangulating our map of coverage before the campaign with nationwide maps of DTP1 and MCV1 coverage produced in previous analyses [24]. For these areas, our map of coverage before and during the campaign (i.e., coverage with at least two doses of MCV) can be used for prioritization to identify areas where lower coverage for MCV2 and higher numbers of children who had not received MCV2 exist following the 2021 measles campaign. Updated maps can also be created when data from more recent campaigns are available.

Considering the ongoing efforts to strengthen the routine immunization programme in Nigeria [45], our analyses can be used to plan, design and implement health facility-based interventions (noting that these analyses also revealed that greater proportions of unvaccinated children during the campaign lived closer to health facilities when using both walking and motorized travel times). Targeting interventions at the health facility level may yield additional benefits over focusing on administrative areas through (i) accounting for the greater variation that exist in the distribution of unvaccinated/zero-dose children within the catchment areas, (ii) strengthening of health facilities to improve the last-mile delivery of RI services and (iii) reaching more zero-dose and under-vaccinated children. Through integration with building footprint data in example catchment areas, we have further demonstrated that our outputs can help with operational activities to identify households that may require outreach services through the RI programme (e.g., those > 2 km from the health facilities). Nevertheless, the catchment areas estimated in our analyses are only operational, since health service utilization patterns are dynamic and will need to be taken into account when defining more robust catchment areas as we further note below. Also, a hybrid approach to catchment area estimation that will involve an automatic delineation using the approach described here and an automatic or manual adjustment of the catchment area boundaries to align with roads and other natural and manmade features on the ground can be adopted to further refine the catchment areas.

Our analyses are subject to some limitations. Areas affected by conflict (e.g., some local government areas in Borno state and the south-eastern part of the country) were excluded from the sampling frame used in the 2021 PCCS [25]; hence the predictions produced by our models are likely more uncertain in these conflict-affected areas. We considered evidence of vaccination from both vaccination cards and maternal/caregiver recall in our work. This could potentially introduce information bias in the analyses. Due to non-availability of ready-to-use population data for children aged 9–59 months, our estimates of un/under-vaccinated children were produced for children aged 12–59 months instead, meaning that we did not include those aged 9–11 months. Also, our zero-dose estimates may be an underestimation if the 2021 PCCS overestimated coverage before and/or after the campaign in some areas (e.g., relative to the 2021 MICS-NICS which was implemented almost at the same time [24,46]). The methodology we used to delineate the catchment areas of the health facilities assumes travel time to the nearest facility, which does not account for bypassing of health facilities, care-seeking behaviour and other user preferences. Other approaches that can overcome these limitations could be used but will require additional detailed datasets to define the catchment areas [47]. We did not account for the uncertainties in the population (and coverage) estimates [48,49,50] when producing the zero-dose estimates. Furthermore, the building footprint data used in our work may be incomplete in some cases. We could not verify whether these were (non-)residential buildings. These potential limitations can be overcome through ground validation of the data or the use of accurate and up-to-date settlement data. Lastly, we could not validate some of the health facilities used in our analyses, particularly in Ebonyi state, to ascertain whether these facilities actually offered vaccination services.

## 5. Conclusions

Nigeria requires an optimal combination of routine and campaign strategies in order to interrupt measles transmission and accelerate progress towards elimination goals. This is particularly essential considering the continued occurrence of measles outbreaks despite repeated vaccination campaigns. Residual zero-dose and under-vaccinated children should be analyzed at the end of vaccination campaigns and all underperforming areas identified should be followed up via appropriate routine immunization strategies. Also, a deep-dive analysis should be undertaken in these underperforming areas to unravel the reasons or risk factors for non-vaccination.

## Data Availability

The data used in this study are available from the National Bureau of Statistics (NBS) Nigeria. Other data such as health facility locations, building footprint data and geospatial covariates are publicly available via the sources referenced in the manuscript. The authors are not allowed to redistribute these datasets.

## Supplementary Materials

The following supporting information can be downloaded at: https://www.mdpi.com/article/10.3390/vaccines12121299/s1, Figure S1: A map showing the 13 states where the 2021 measles campaign was implemented. Figure S2: Distribution of cluster level sample sizes for all six indicators. Table S1: Covariates selected to model vaccination coverage in the study. Figure S3: Covariates used to model vaccination coverage in the study. Figure S4: Locations of all (left panels) and selected (right panels) health facilities in Kano and Ebonyi states included in the study. Figure S5: Violin plots showing the distribution of modelled estimates of PCCS indicators for each state. Table S2: Modelled estimates of 2021 measles post-campaign coverage survey indicators at the state level. Figure S6: Modelled estimates of coverage among MCV zero-dose children at the local government area (LGA) level. Figure S7: Distribution of unvaccinated children before and during the SIA within different walking and motorised travel time bands in Kano state. Figure S8: Distribution of unvaccinated children before and during the SIA within different walking and motorised travel time bands in Ebonyi state. Figure S9: Comparisons between 2021 PCCS modelled estimates and direct survey estimates at the state level. Figure S10: A map of an enumeration area within Yamma 2 ward in Katsina State overlaid with Google building footprint data to identify the buildings within the area. The enumeration area was created using the preEA tool referenced in the manuscript.
