# Supplementary material for: Geospatial Variation in Vaccination Coverage and Zero-Dose Prevalence at the District, Ward and Health Facility Levels Before and After a Measles Vaccination Campaign in Nigeria
Source: Vaccines (Basel). 2024 Nov 21;12(12):1299. doi: 10.3390/vaccines12121299 (PMC11680329; doi:10.3390/vaccines12121299)
Supplement: Supplementary file 1 [file vaccines-12-01299-s001.zip › vaccines-3269231-supplementary.pdf]

Geospatial variation in vaccination coverage and zero-dose prevalence at the district, ward and health facility levels before and after a measles vaccination campaign in Nigeria

C. Edson Utazi[1,2], Iyanuloluwa D. Olowe[1], Theo H. M. Chan[3], Winfred Dotse-Gborgortsi[1], John Wagai[4], Jamila A. Umar[5], Sulaiman Etamesor[5], Brian Atuhaire[6], Biyi Fafunmi[7], Jessica Crawford[6], Adeyemi Adeniran[7], Andrew J. Tatem[1]

[1] *WorldPop, School of Geography and Environmental Science, University of Southampton, Southampton, SO17 1BJ, UK*
[2] *Department of Statistics, Nnamdi Azikiwe University, Awka, PMB 5025, Nigeria*
[3]*School of Mathematical Sciences, University of Southampton, Southampton, SO17 1BJ, UK*
[4]*World Health Organization Consultant, Abuja, Nigeria*
[5]*National Primary Health Care Development Agency, Abuja, Nigeria*
[6]*Gavi, The Vaccine Alliance, Geneva, Switzerland*
[7]*National Bureau of Statistics, Abuja, Nigeria*

Supplementary File

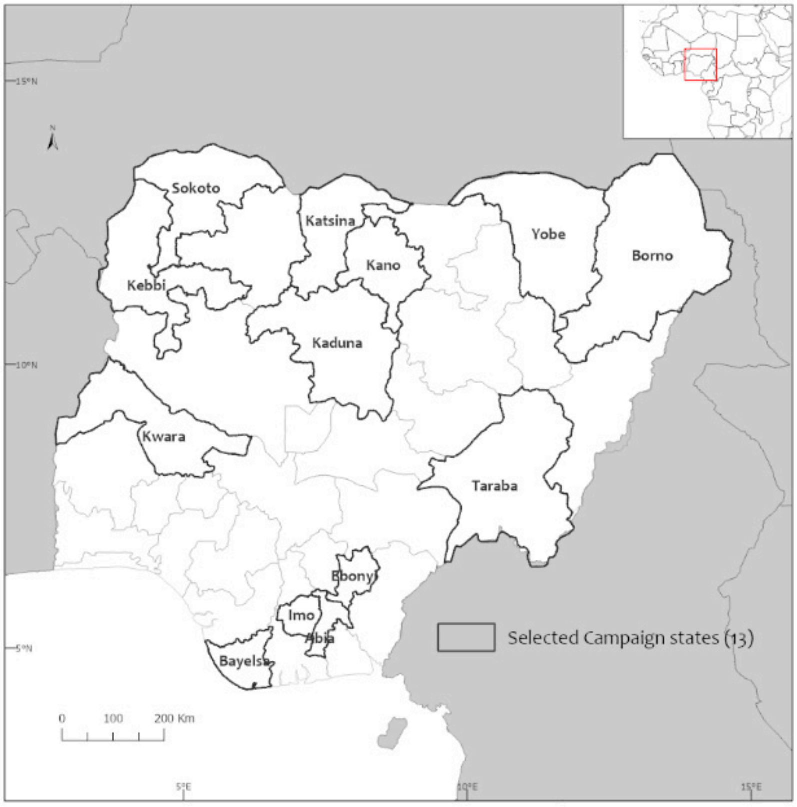

Supplementary Figure S1: A map showing the 13 states where the 2021 measles campaign was implemented.

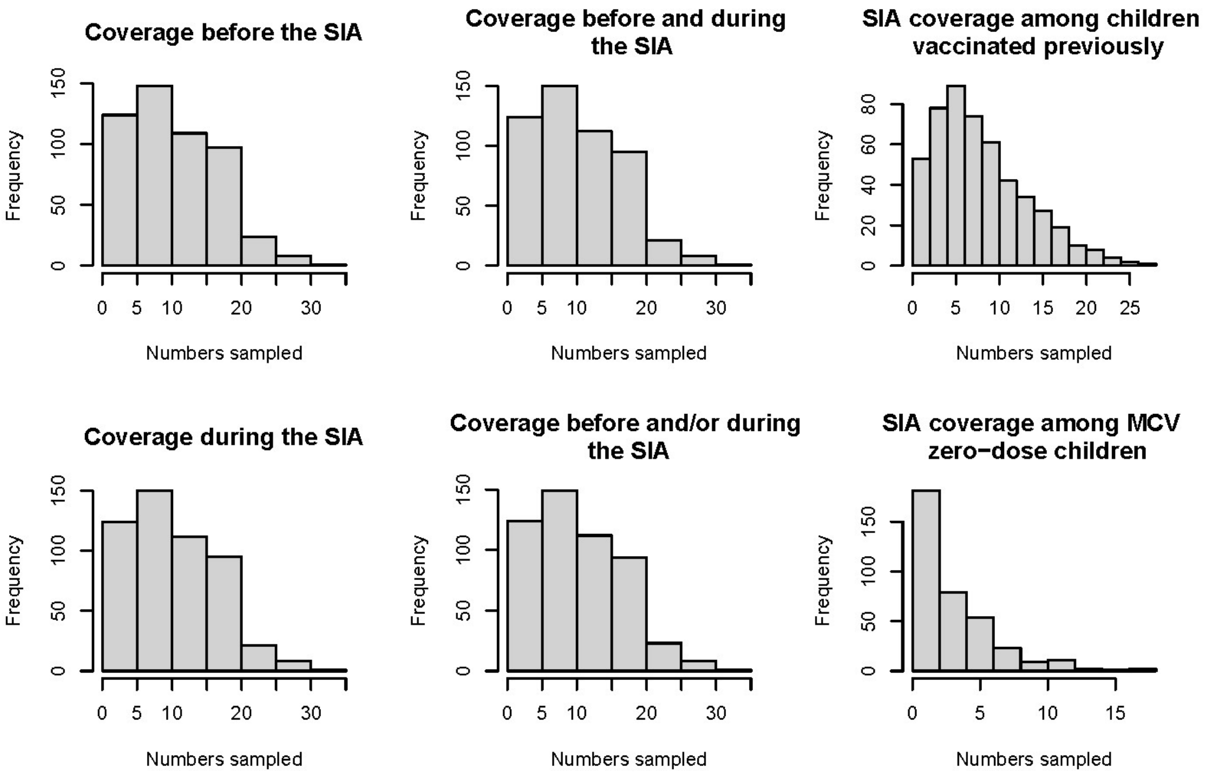

Supplementary Figure S2: Distribution of cluster level sample sizes for all six indicators.

Supplementary Table S1: Covariates selected to model vaccination coverage in the study.

Geospatial covariate	Definition	Spatial Resolution	Source
Distance to UCDP Conflict Locations	Distance to conflict areas identified by the Uppsala Conflict Data Program (UCDP)	1km	Pettersson, Therese, Shawn Davis, Amber Deniz, Garoun Engström, Nanar Hawach, Stina Högbladh, Margareta Sollenberg & Magnus Öberg (2021). Organized violence 1989-2020, with a special emphasis on Syria. Journal of Peace Research 58(4). Sundberg, Ralph and Erik Melander (2013) Introducing the UCDP Georeferenced Event Dataset. Journal of Peace Research 50(4). https://ucdp.uu.se/downloads/index.html#ged_global
Livestock Index	Count of livestock per square kilometre	1km	Gilbert M., G. Nicolas, G. Cinardi, G.R.W. Wint, S. Vanwambeke, T.P. Robinson (2018) Global Distribution Data for Cattle, Buffaloes, Horses, Sheep, Goats, Pigs, Chickens and Ducks in 2010. Sci. Data. 5:180227 https://doi.org/10.1038/sdata.2018.227
Night time	Annual Visible	1km	C. D. Elvidge, K. E. Baugh, M. Zhizhin, and F.-C. Hsu,

Lights	Infrared Imaging Radiometer Suite (VIIRS) nighttime lights		"Why VIIRS data are superior to DMSP for mapping nighttime lights," Asia-Pacific Advanced Network 35, vol. 35, p. 62, 2013
Distance to all OSM roads	Distance to all road types identified by OpenStreetMap	1km	WorldPop (www.worldpop.org - School of Geography and Environmental Science, University of Southampton; Department of Geography and Geosciences, University of Louisville; Departement de Geographie, Universite de Namur) and Center for International Earth Science Information Network (CIESIN), Columbia University (2018). Global High Resolution Population Denominators Project - Funded by The Bill and Melinda Gates Foundation (OPP1134076). https://dx.doi.org/10.5258/SOTON/WP00644
Average Maximum Temperature	Maximum Temperature averaged between 2017 and 2021	1km	Harris I, Osborn TJ, Jones P and Lister D (2020) Version 4 of the CRU TS Monthly High-Resolution Gridded Multivariate Climate Dataset. Sci Data 7,, 109 (2020). https://doi.org/10.1038/s41597-020-0453-3
Distance to Cultivated areas	Distance to edges of cultivated areas from ESA-CCI-LC classes.	1km	WorldPop (www.worldpop.org - School of Geography and Environmental Science, University of Southampton; Department of Geography and Geosciences, University of Louisville; Departement de Geographie, Universite de Namur) and Center for International Earth Science Information Network (CIESIN), Columbia University (2018). Global High Resolution Population Denominators Project - Funded by The Bill and Melinda Gates Foundation (OPP1134076). https://dx.doi.org/10.5258/SOTON/WP00644
Elevation	Topography	1km	WorldPop (www.worldpop.org - School of Geography and Environmental Science, University of Southampton; Department of Geography and Geosciences, University of Louisville; Departement de Geographie, Universite de Namur) and Center for International Earth Science Information Network (CIESIN), Columbia University (2018). Global High Resolution Population Denominators Project - Funded by The Bill and Melinda Gates Foundation (OPP1134076). https://dx.doi.org/10.5258/SOTON/WP00644
Distance to GHSL settlements	Distance to settlements identified in the Global Human Settlement Layer dataset	1km	WorldPop (www.worldpop.org - School of Geography and Environmental Science, University of Southampton; Department of Geography and Geosciences, University of Louisville; Departement de Geographie, Universite de Namur) and Center for International Earth Science Information Network (CIESIN), Columbia University (2018). Global High Resolution Population Denominators Project - Funded by The Bill and Melinda Gates Foundation (OPP1134076).

			https://dx.doi.org/10.5258/SOTON/WP00644
Average Malaria Prevalence	Parasite rate in 2-10 year olds, averaged between 2015 and 2019	1km	Weiss DJ, Lucas TCD, Nguyen M, et al. Mapping the global prevalence, incidence, and mortality of Plasmodium falciparum, 2000–17: a spatial and temporal modelling study. Lancet 2019; published online June 19. DOI: 10.1016/S0140-6736(19)31097-9. Battle KE, Lucas TCD, Nguyen M, et al. Mapping the global endemicity and clinical burden of Plasmodium vivax, 2000–17: a spatial and temporal modelling study. Lancet 2019; published online June 19. DOI: 10.1016/S0140-6736(19)31096-7
Walking Travel time to Health Facilities	Travel time (by walking distance) to health facilities	1km	D.J. Weiss, A. Nelson, C.A. Vargas-Ruiz, K. Gligoric?, S. Bavadekar, E. Gabrilovich, A. Bertozzi-Villa, J. Rozier, H.S. Gibson, T. Shekel, C. Kamath, A. Lieber, K. Schulman, Y. Shao, V. Qarkaxhija, A.K. Nandi, S.H. Keddie, S. Rumisha, P. Amratia, R. Arambepola, E.G. Chestnutt, J.J. Millar, T.L. Symons, E. Cameron, K.E. Battle, S. Bhatt, and P.W. Gething. Global maps of travel time to healthcare facilities. (2020). Nature Medicine. doi:10.1038/s41591-020-1059-1 Available from: https://malariaatlas.org/research-project/accessibility-to-healthcare/

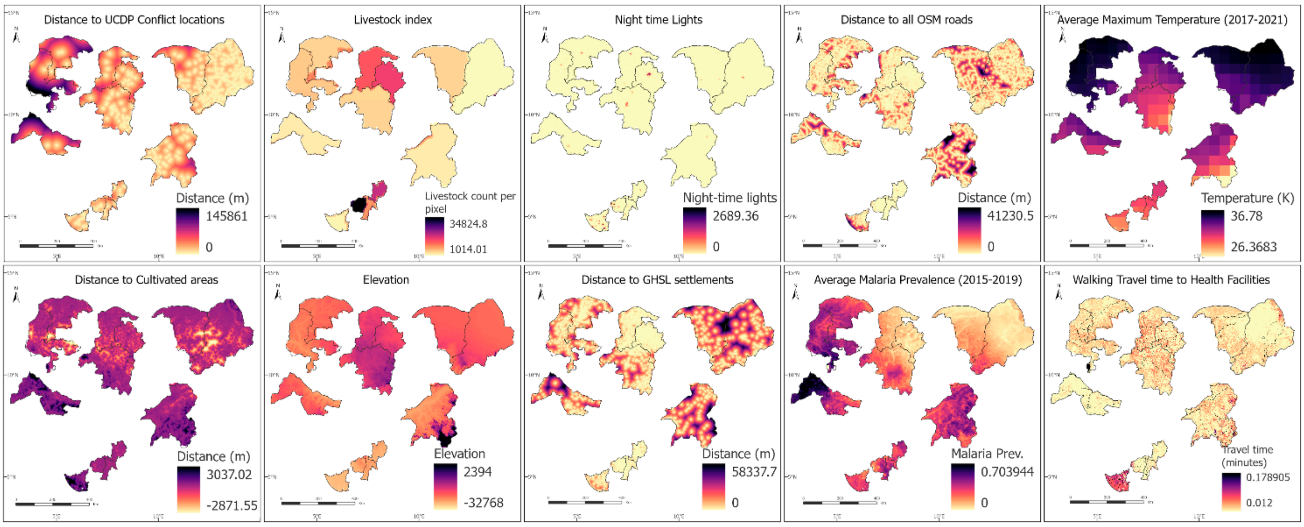

Supplementary Figure S3: Covariates used to model vaccination coverage in the study.

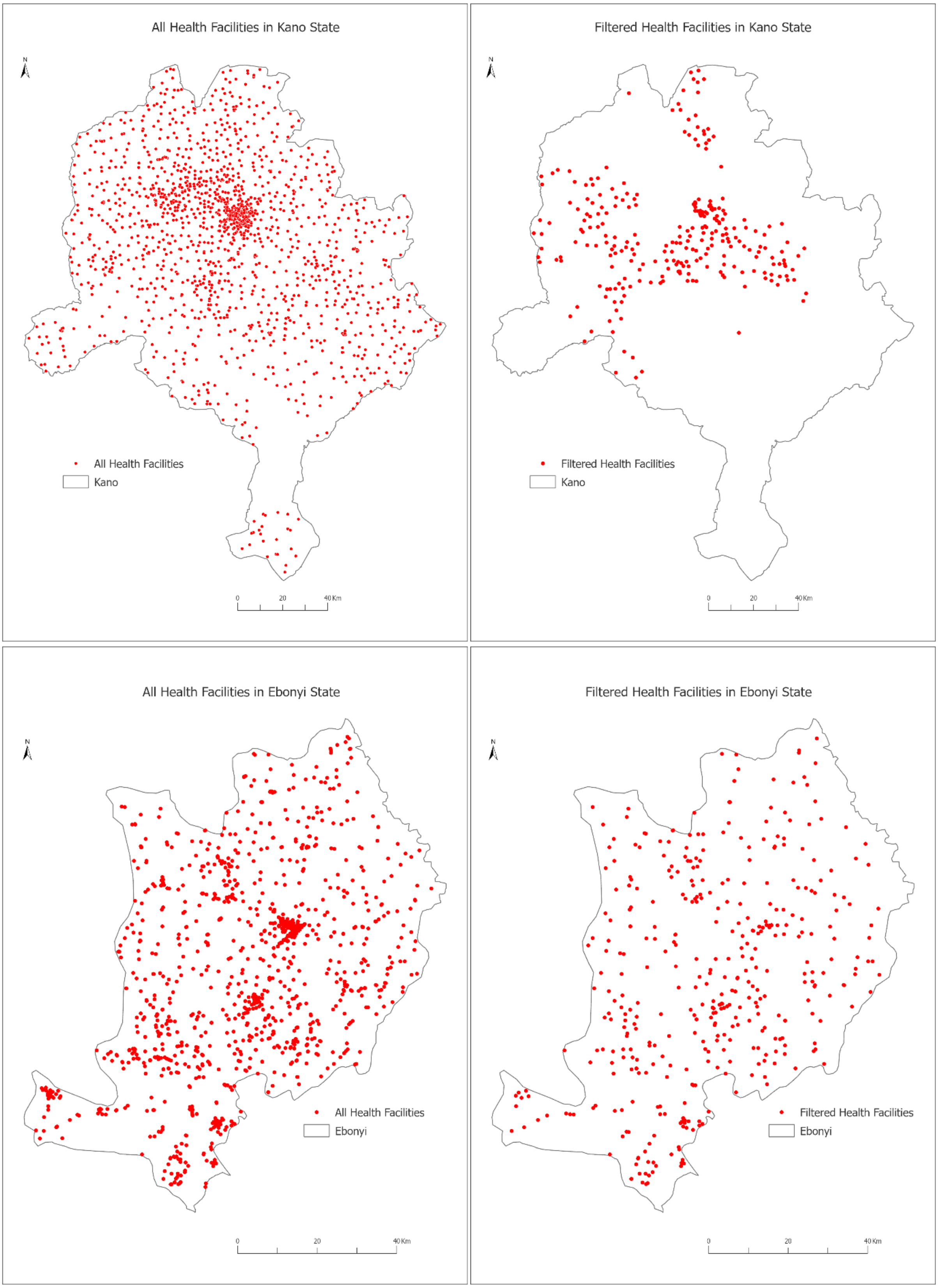

Supplementary Figure S4: Locations of all (left panels) and selected (right panels) health facilities in Kano and Ebonyi states included in the study.

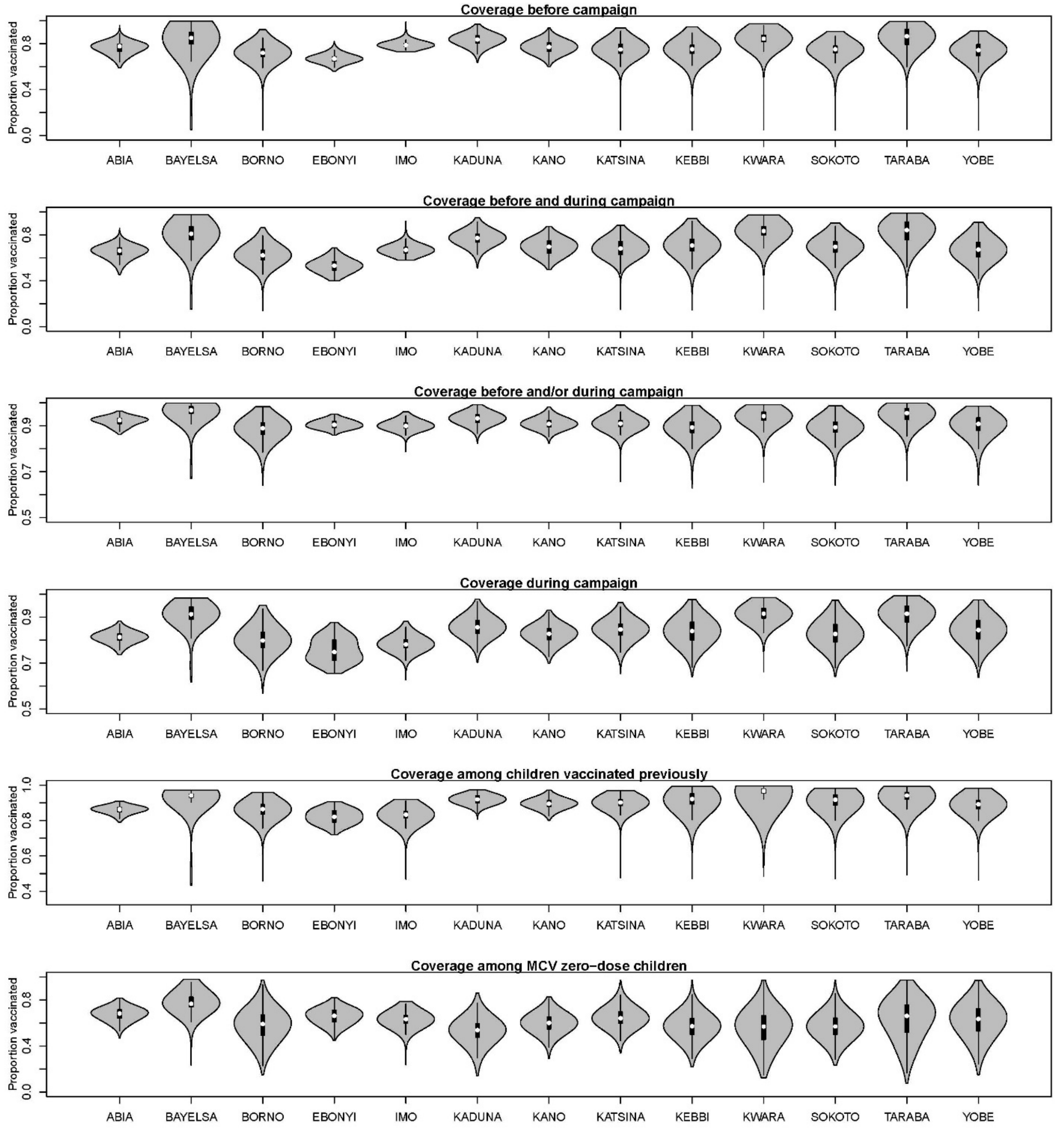

Supplementary Figure S5: Violin plots showing the distribution of modelled estimates of PCCS indicators for each state.

Supplementary Table S2: Modelled estimates of 2021 measles post-campaign coverage survey indicators at the state level

State	2021 estimate of children aged 1-4 years	Coverage before the campaign	Coverage before and during the campaign	Coverage during the campaign	Coverage among children vaccinated previously	Coverage among MCV zero-dose children	Coverage before and/or during the campaign
Abia	375835	0.7768	0.6597	0.8082	0.8481	0.6882	0.9230
Bayelsa	247057	0.7728	0.7327	0.8526	0.8833	0.7191	0.9100
Borno	987788	0.7107	0.5925	0.7537	0.8098	0.5752	0.8640
Ebonyi	380362	0.6525	0.5086	0.7389	0.8024	0.6492	0.8930
Imo	551770	0.7816	0.6656	0.7702	0.8236	0.5976	0.8820
Kaduna	1405201	0.8401	0.7619	0.8421	0.9049	0.5186	0.9330
Kano	2240534	0.7846	0.6941	0.8112	0.8750	0.5678	0.9040
Katsina	1429811	0.7276	0.6585	0.8392	0.8913	0.6469	0.8970
Kebbi	747121	0.7343	0.6800	0.8223	0.8993	0.5753	0.8850
Kwara	469823	0.8162	0.7831	0.8822	0.9296	0.6192	0.9220
Sokoto	901541	0.7510	0.6848	0.8050	0.8974	0.5405	0.8810
Taraba	484847	0.8156	0.7687	0.8725	0.9105	0.6324	0.9290
Yobe	554201	0.7380	0.6621	0.8268	0.8778	0.6274	0.8970

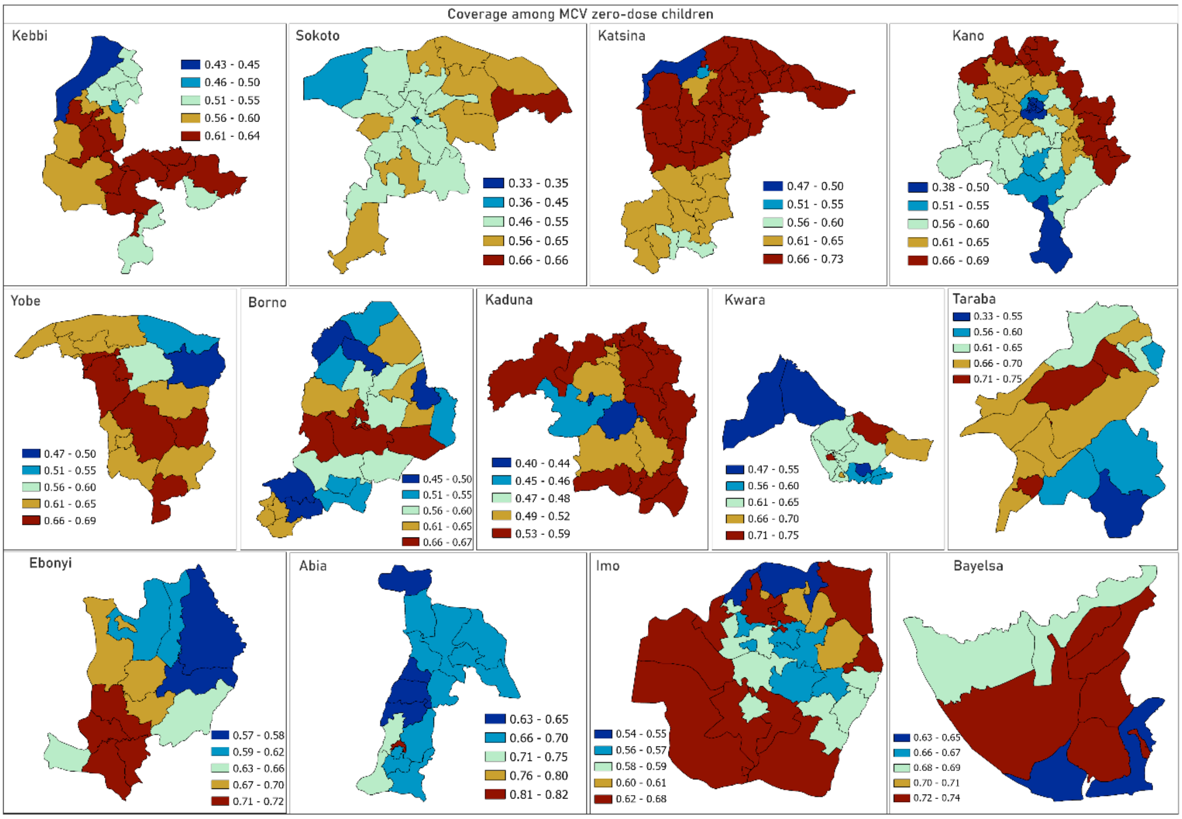

Supplementary Figure S6: Modelled estimates of coverage among MCV zero-dose children at the local government area (LGA) level.

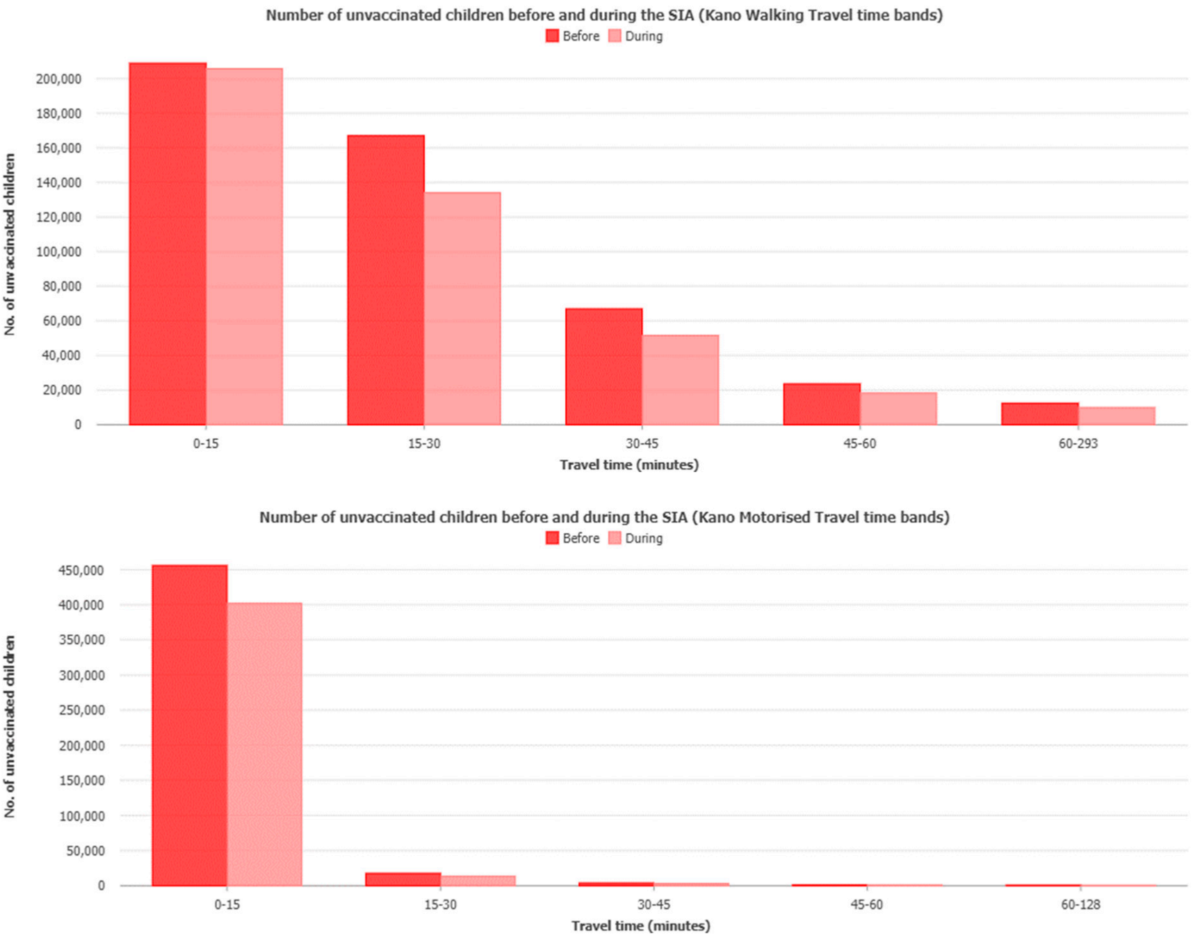

Supplementary Figure S7: Distribution of unvaccinated children before and during the SIA within different walking and motorised travel time bands in Kano state.

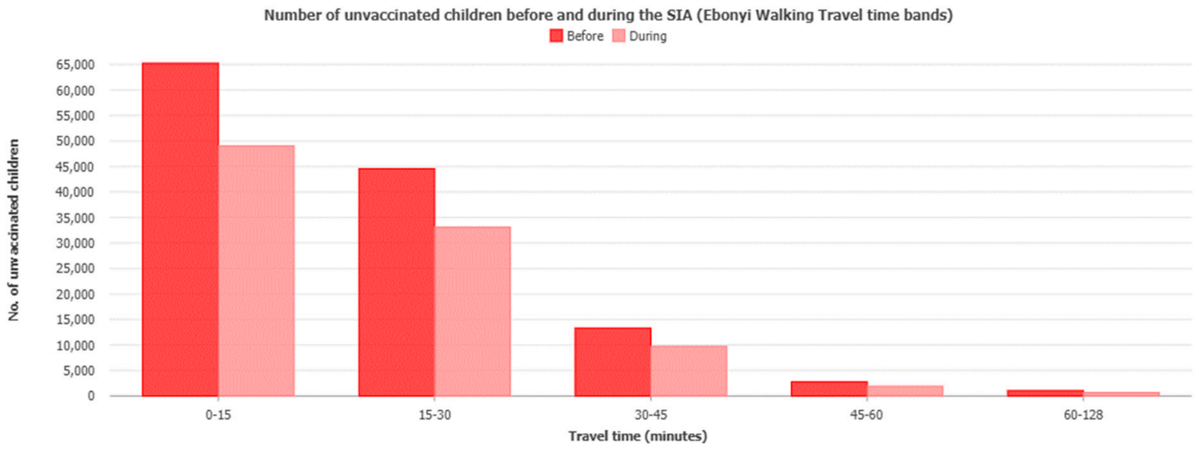

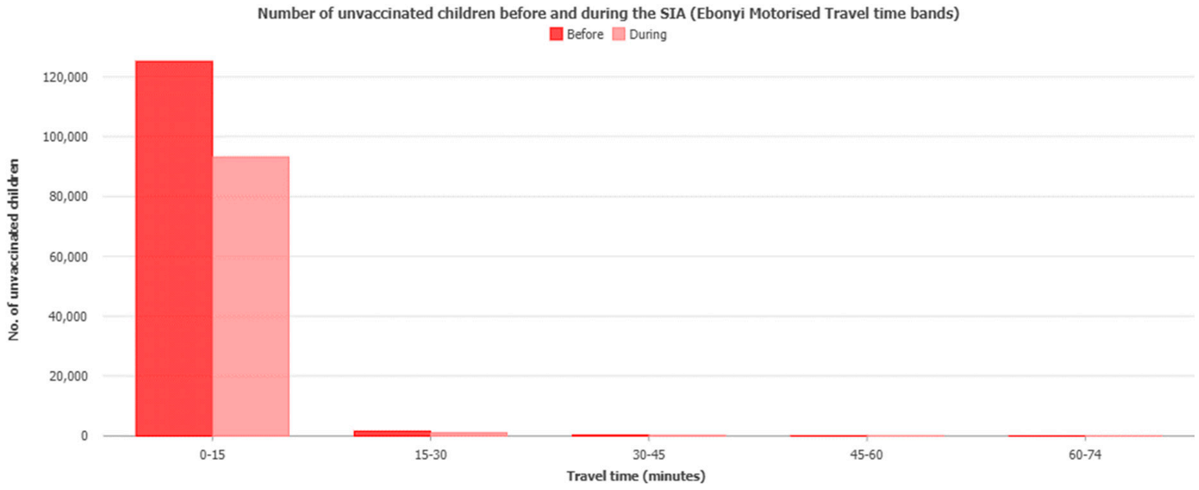

Supplementary Figure S8: Distribution of unvaccinated children before and during the SIA within different walking and motorised travel time bands in Ebonyi state.

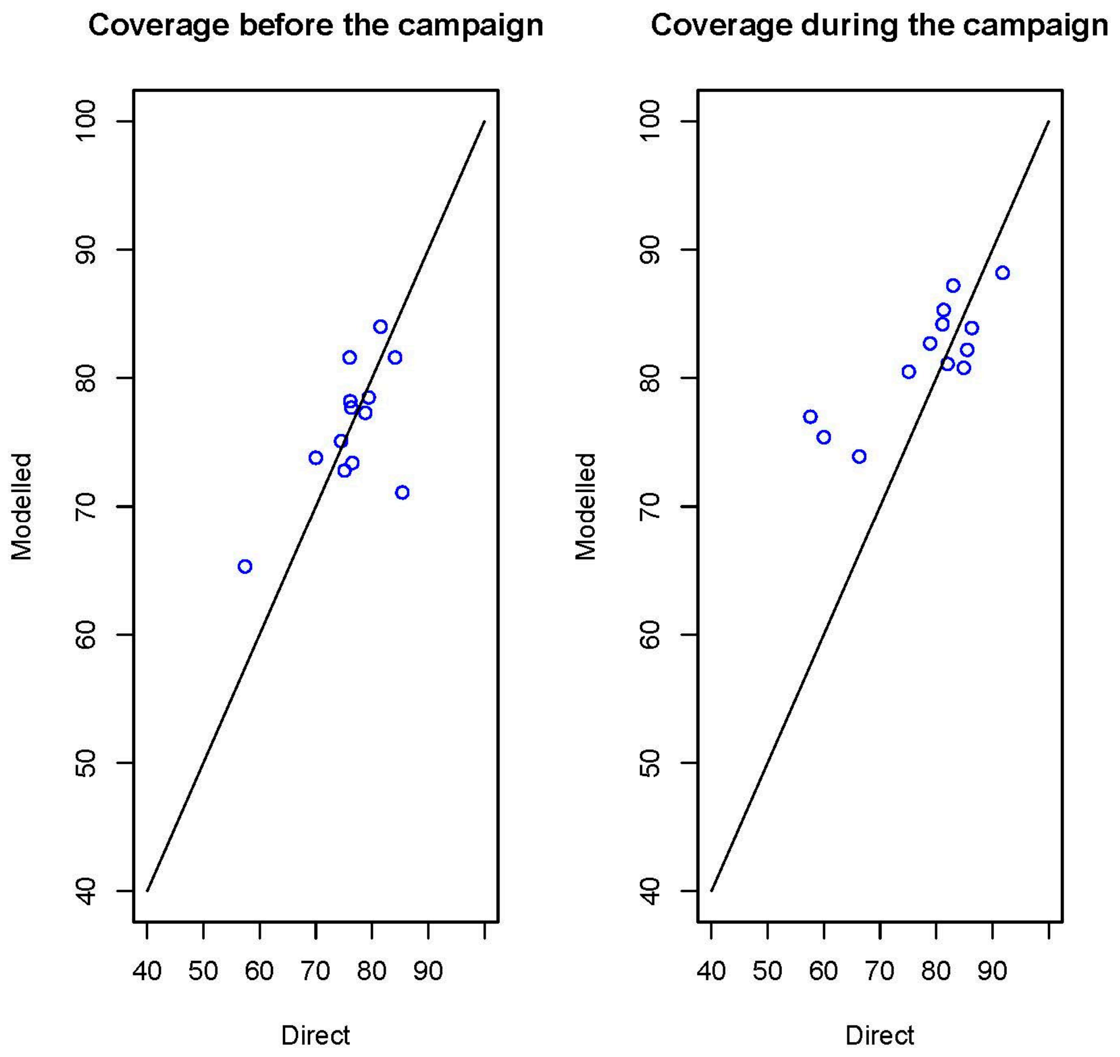

Supplementary Figure S9: Comparisons between 2021 PCCS modelled estimates and direct survey estimates at the state level.

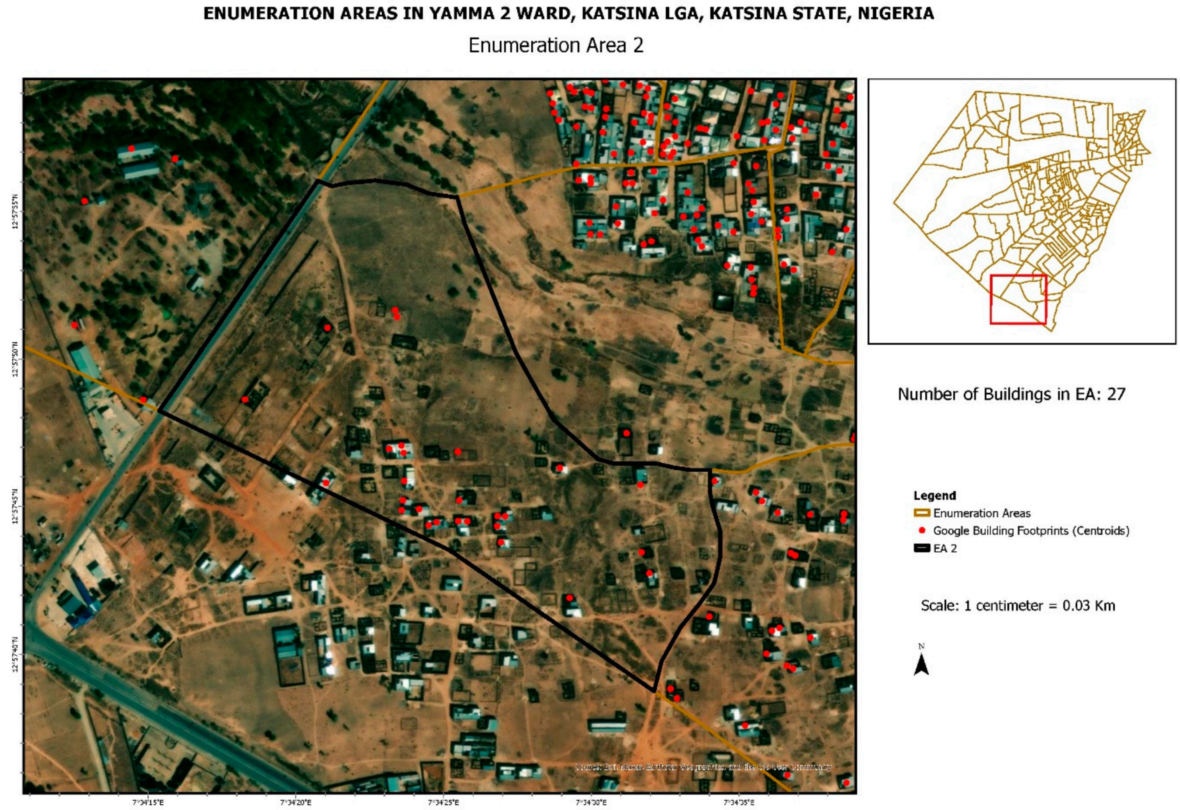

Supplementary Figure S10: A map of an enumeration area within Yamma 2 ward in Katsina State overlaid with Google building footprint data to identify the buildings within the area. The enumeration area was created using the preEA tool referenced in the manuscript.
